# Hospitalization cost trends for patients with impacted teeth: a double-breakpoint interrupted time series analysis

**DOI:** 10.3389/fpubh.2026.1875608

**Published:** 2026-07-24

**Authors:** Ni An, Liangmei Jiang, Yuanbo Song, Ran Xiang, Jinpeng Zhang, Yiyang Wang, Fengming Li

**Affiliations:** 1 School of Management, Shandong Second Medical University, Weifang, Shandong, China; 2 Operation Management Department, Linyi People's Hospital, Linyi, Shandong, China

**Keywords:** clinical pathway management, DRG payment, hospitalization costs, impacted teeth, interrupted time series analysis

## Abstract

**Objective:**

To evaluate the impact of Diagnosis-Related Group (DRG) payment reform and clinical pathway management on the hospitalization costs and cost structures for patients with impacted teeth.

**Methods:**

We analyzed admission data for 1,206 patients with impacted teeth at a Grade A tertiary public hospital in City L from September 1, 2021, to December 31, 2024. A double-breakpoint interrupted time series analysis was employed to assess level and trend changes in total and component costs. Intervention points were set at August 1, 2022 (DRG reform), and September 1, 2023 (clinical pathway management).

**Results:**

Prior to the DRG reform, consumable costs trended upward while nursing costs trended downward (*p* < 0.05). After the DRG reform, nursing costs transitioned to a significant increasing trend (*p* < 0.05). Following the implementation of clinical pathway management, significant decreasing trends were observed across total hospitalization costs, consumable costs, treatment costs, nursing costs, general medical service costs, total insurance payments, and out-of-pocket expenses (*p* < 0.05).

**Conclusion:**

DRG reform alone was not associated with a significant reduction in hospitalization costs for patients with impacted teeth during the initial implementation phase. However, when integrated with clinical pathway management, the results suggested an association between the combined implementation and optimized cost structure as well as reduced hospital stays. Given the limitations of a single-center design and quasi-experimental design, these findings should be interpreted as associational evidence, and further research is warranted to validate its impact on clinical quality.

## 1 Introduction

Impacted teeth represent a common dental condition characterized by teeth remaining trapped within the jawbone, failing to erupt during the physiological eruption period. This condition can result in incomplete dentition and malocclusion, compromising facial aesthetics and, consequently, patient mental health and quality of life (1). Because dental treatment is specialized, time-sensitive, and longitudinal, it imposes some financial burden on patients (2). Diagnosis-Related Groups (DRGs) function as a payment system that categorizes short-stay inpatients based on diagnoses, surgical procedures, and complications or comorbidities. As a tool for refining the management of healthcare services, the DRG system reflects disease severity, resource utilization, and prognosis (3). While it facilitates cost control and resource allocation in public hospitals, a singular focus on cost containment may jeopardize clinical quality (4). Conversely, clinical pathway management aims to enhance clinical quality and efficiency while reducing expenditures (5). Theoretically, the combined implementation of these two policies may balance between cost control and efficiency improvement; however, empirical evidence regarding their combined effects in oral surgery single-disease populations remains scarce.

While existing literature extensively examines the overall costs of oral diseases (6), the economic burden of hospital-acquired infections (7), and inpatient costs for oral tumors (8, 9), there remains a notable lack of research evaluating the policy effectiveness concerning impacted teeth. Therefore, this study utilizes data from patients with a primary diagnosis of impacted teeth at a Grade A tertiary public hospital in City L as a case study. By identifying the implementation of DRG payment reform and clinical pathway management as intervention points, we systematically evaluate dynamic changes in total inpatient costs. Our objective is to provide a scientific basis for the refined management of specific dental conditions and the mitigation of patient financial burdens.

## 2 Materials and methods

### 2.1 Data sources

Data for this study were retrieved from medical record front sheets and medical insurance settlement lists at a tertiary general hospital in City L, covering the period from September 1, 2021, to December 31, 2024. We matched these records to identify patients with a primary diagnosis of impacted tooth (ICD-10: K01.000) and a primary procedure of “extraction of impacted tooth with flap reflection” (ICD-9-CM-3: 23.1900 × 006). After excluding patients who withdrew from the clinical pathway and cases with incomplete or missing data, 1,206 patients were included in the final analysis.

Collected variables included patient demographics (including sex and age), operational efficiency indicators (actual length of stay, preoperative length of stay, and number of hospitalizations), and hospitalization costs (total costs and subcategories: medication, consumables, treatment, diagnosis, nursing, and general medical services). We also analyzed payer composition through total insurance payments and out-of-pocket expenses. In this study, the sum of total insurance payments and out-of-pocket expenses constituted the total hospitalization costs. To ensure comparability across time periods, we adjusted all cost data to the 2024 price level using the healthcare consumer price index (CPI) for the province where City L is located, as published by the National Bureau of Statistics of China, with 2024 as the base year.

In this study, the majority of hospitalized patients undergoing impacted tooth extraction were children and adolescents aged 18 years and younger, accounting for over 94% of the cohort. The impacted teeth in this population were predominantly supernumerary teeth located in the maxillary anterior region, which were most frequently detected during dental visits due to malocclusion or delayed eruption of permanent teeth during the mixed dentition period. Given their young age, low intraoperative compliance, and the fact that the impacted teeth were often deeply positioned requiring bone removal during extraction, the surgical procedures were technically demanding. Consequently, all these patients underwent extraction under general anesthesia in an inpatient setting. In contrast, adult patients with impacted mandibular third molars that were relatively superficially positioned and without specific risks were generally extracted under local anesthesia in an outpatient setting, and were therefore less likely to be included in the inpatient sample. Thus, the findings of this study primarily reflect cost trends for the pediatric and adolescent population undergoing inpatient extraction of impacted teeth under general anesthesia, and extrapolation to adult outpatients or those undergoing extraction under local anesthesia should be made with caution.

### 2.2 Research methods

Statistical analyses were performed using Stata 18.0. Skewed continuous variables are presented as medians and interquartile ranges (*M*[*P*_25_, *P*_75_]). We utilized the Kruskal–Wallis H rank-sum test for multi-group comparisons and the rank-sum test for pairwise comparisons. Categorical variables are presented as relative frequencies and were compared using the *χ^2^* test. Statistical significance was set at *p* < 0.05.

Interrupted time series (ITS) analysis evaluates the actual effectiveness of interventions by assessing changes in the level and trend of outcome indicators before and after the intervention. This method can evaluate both immediate effects and long-term trends, and has been widely used in health policy evaluation (10–12). we employed an ITS analysis with a complete time series comprising 40 monthly data points. August 1, 2022, was designated as the intervention point for the DRG payment reform. The pre-reform period spanned from September 1, 2021, to July 31, 2022, covering 11 data points and 157 patients. The post-reform period extended from August 1, 2022, to August 31, 2023, covering 13 data points and 438 patients. September 1, 2023, marked the implementation of clinical pathway management under the DRG system, with the subsequent phase extending through December 31, 2024, covering 16 data points and 611 patients. The independent variable was defined as time, with month as the unit of analysis. For each cost category, we calculated the sum of costs for all included patients in that month and divided it by the number of visits in that month to obtain the average cost per visit, which served as the dependent variable in the ITS model.

The following ITS model was established:

*Y*_t_ = *β*_0_ + *β_1_ T* + *β_2_X_1_* + *β_3_ TX_1_* + *β_4_X_2_* + *β_5_ TX_2_* + *ε*,

where *Y_t_* represents the outcome variable at time *t*; *T* is the continuous variable (months); *X*_1_ and *X*_2_ are binary dummy variables for DRG reform and clinical pathway implementation, respectively; and *TX*_1_ and *TX*_2_ represent the slopes following DRG reform and clinical pathway implementation, respectively. In this model, *β*_0_ represents the baseline level, and *β*_1_ indicates the pre-intervention trend. The coefficients *β_2_* and *β_4_* represent immediate level changes following the DRG reform and clinical pathway management, respectively. Similarly, *β_3_* and *β_5_* represent trend changes (the difference in slopes) attributable to each respective intervention. Specifically, *β_1_* + *β_3_* represents the slope of inpatient costs per unit of time following the DRG reform, while *β_1_* + *β_3_* + *β_5_* represents the slope following clinical pathway implementation. Finally, *ε* represents the error term (13). The Cumby-Huizinga test for autocorrelation was performed for each outcome variable, with the maximum lag order set to 6. For outcome variables with evidence of autocorrelation, Newey-West standard errors with the corresponding lag order (1 to 6) were applied to correct for heteroskedasticity and autocorrelation. For outcome variables without evidence of autocorrelation (that is, non-significant at lag 1), no Newey-West adjustment was made.

## 3 Results

### 3.1 Demographic characteristics

The study cohort comprised 1,206 patients: 157 in the pre-DRG period, 438 in the post-DRG period, and 611 following the integration of clinical pathway management. Comparative analysis indicated no statistically significant differences in sex, age distribution, or insurance type across the three study phases (*p* > 0.05), confirming baseline cohort comparability (Table 1).

**Table 1:** Demographic characteristics of patients with impacted teeth (*n*, %).

Characteristic	Before the DRG payment	After the DRG payment reform	After the implementation of clinical pathway management	*χ^2^*	*p*
Sex				0.7924	0.673
Men	119(75.80)	316(72.15)	445(72.83)		
Women	38(24.20)	122(27.85)	166(27.17)		
Age				1.0477	0.592
≤18	148(94.27)	411(93.84)	582(95.25)		
>18	9(5.73)	27(6.16)	29(4.75)		
Insurance type				1.3401	0.512
Resident	148(94.27)	422(96.35)	587(96.07)		
Employee	9(5.73)	16(3.65)	24(3.93)		

### 3.2 Analysis of operational efficiency indicators

Across all three phases, the median number of hospitalizations remained constant at 1, with no statistically significant difference (*p* > 0.05). However, operational efficiency metrics improved significantly following the integration of clinical pathway management. The median actual length of stay before the DRG reform, after the DRG payment reform, and after the implementation of clinical pathway management was 4, 4, and 1, respectively. Similarly, the median preoperative length of stay across the same three periods was 2, 2, and 0, respectively. These differences in length of stay and preoperative duration were all statistically significant (*p* < 0.05), indicating that clinical pathway management significantly optimized hospital throughput (Table 2).

**Table 2:** Operational efficiency indicators under DRG payment reform and clinical pathway management (*M* [*P*_25_, *P*_75_]).

Operational efficiency indicator	Before the DRG payment reform	After the DRG payment reform	After the implementation of clinical pathway management	*Z*	*p*
Actual length of hospital stay	4 (4, 5)	4(3, 4)	1(1, 2)	661.565	<0.001
Number of hospitalizations	1 (1, 1)	1 (1, 1)	1 (1, 1)	0.295	0.863
Number of days spent in the hospital prior to surgery	2 (2, 2)	2 (1, 2)	0 (0, 1)	496.580	<0.001

### 3.3 Analysis of inpatient cost structures

Table 3 presents the median values and intergroup comparisons for each cost category across the three phases. Following the DRG reform, total hospitalization costs, consumable costs, treatment costs, medication costs, and total insurance payments increased by 613.49, 104.53, 486.39, 32.48 and 580.69 yuan, respectively. Conversely, diagnosis and general medical service costs decreased by 91.59 and 22.91 yuan, respectively (*p* < 0.05). Following the implementation of clinical pathway management in the post-DRG reform period, significant reductions were observed across several expenditure categories (*p* < 0.05). Specifically, total hospitalization costs decreased by 840.21 yuan, followed by reductions in out-of-pocket expenses (491.44 yuan), total insurance payments (337.14 yuan), and treatment costs (205.53, yuan). Additionally, costs for diagnosis, medication costs, general medical services, consumables, and nursing fell by 205.02, 203.92, 185.66, 129.72, and 64.90 yuan, respectively. Compared with the pre-DRG reform period, clinical pathway management led to significant reductions in several key areas (*p* < 0.05). Specifically, out-of-pocket expenses and diagnosis costs decreased by 515.81 and 296.61 yuan, respectively. Notable savings were also observed in costs for total hospitalization (138.53 yuan), general medical services (208.57 yuan), medications (171.44 yuan), and nursing (54.83 yuan). Conversely, treatment costs and total insurance payments saw significant increases of 280.86 and 243.55 yuan, respectively.

**Table 3:** Analysis of inpatient hospitalization costs (*M* [*P*_25_, *P*_75_]).

Cost items	Before the DRG payment reform	After the DRG payment reform	After the implementation of clinical pathway management	*Z*	*P*
Total hospitalization costs	5311.24 (4806.29, 6015.22)	5924.73 (5351.42, 7014.38)^a^	5084.52 (4491.46, 6124.11)^ab^	144.957	<0.001
Consumable costs	922.15 (817.31, 1038.50)	1026.68 (923.11, 1700.19)^a^	896.96 (787.90, 1006.12)^b^	146.408	<0.001
Treatment costs	2567.86 (2176.79, 3031.33)	3054.25 (2486.45, 3491.82)^a^	2848.72 (2450.08, 3371.93)^ab^	38.938	<0.001
Diagnosis costs	822.73 (628.87, 1032.19)	731.14 (603.50, 834.15)^a^	526.12 (523.50, 727.50)^ab^	314.784	<0.001
Medication costs	555.67 (483.82, 655.71)	588.15 (496.24, 710.11)^a^	384.23 (304.59, 484.63)^ab^	371.815	<0.001
Nursing costs	118.83(118.83, 136.95)	128.90 (116.58, 148.74)	64.00 (56.00, 84.00)^ab^	573.98	<0.001
General medical service costs	296.67 (237.60, 318.07)	273.76 (235.77, 337.60)^a^	88.10 (68.10, 176.20)^ab^	609.019	<0.001
Total insurance payments	1978.61 (1711.71, 2291.56)	2559.30 (2207.90, 2986.63)^a^	2222.16 (1934.32, 2587.66)^ab^	157.334	<0.001
Out-of-pocket expenses	3324.32 (3077.38, 3705.35)	3299.95 (3068.51, 3887.59)	2808.51 (2535.92, 3279.16)^ab^	211.643	<0.001

^a^:*p* < 0.05 compared with the pre-DRG period; ^b^:*p* < 0.05 compared with the post-DRG period.

### 3.4 ITS analysis of inpatient costs across policy implementation stages

**Figure 1:**
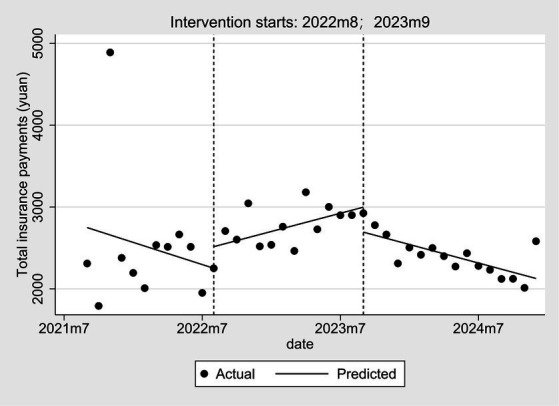
Trends in total insurance payments.

**Figure 2:**
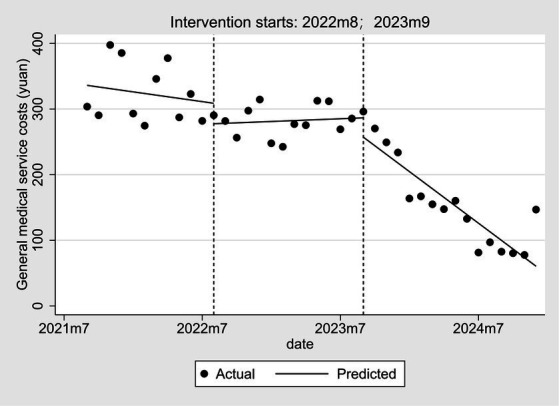
Trends in general medical service costs.

**Figure 3:**
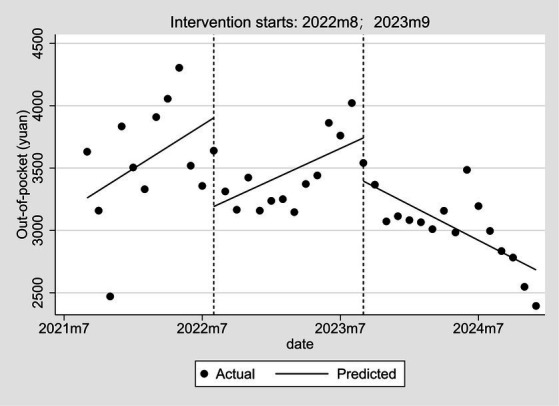
Trends in out-of-pocket expenses.

**Figure 4:**
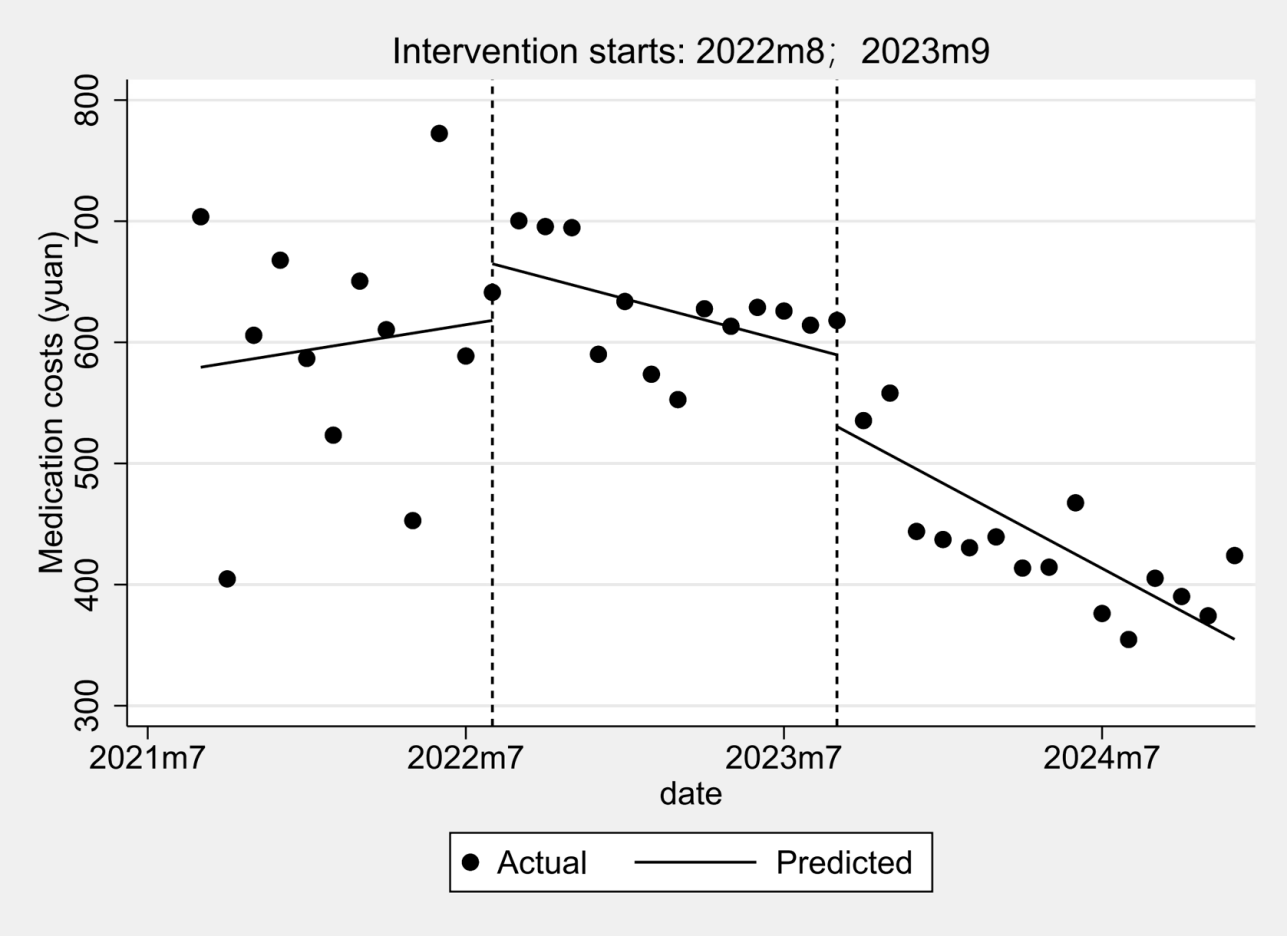
Trends in medication costs.

**Figure 5:**
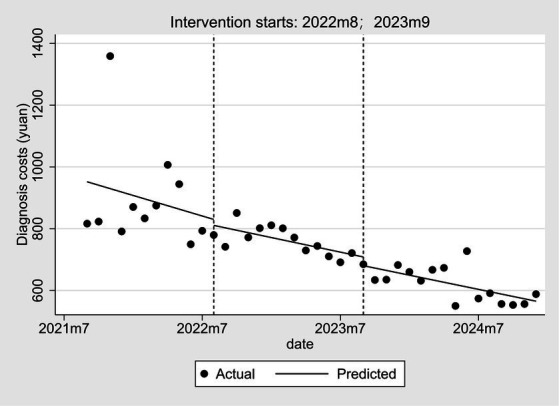
Trends in diagnosis costs.

**Figure 6:**
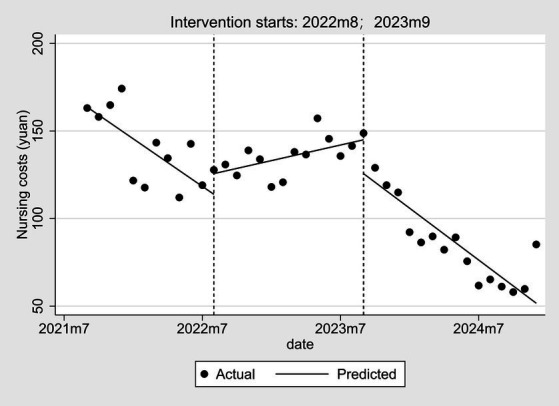
Trends in nursing costs.

**Figure 7:**
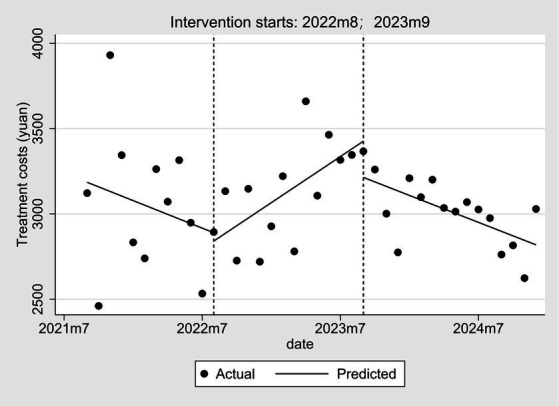
Trends in treatment costs.

The ITS analysis estimated the monthly trends in total hospitalization costs and their various components, as shown in Table 4 and Figures 1–7. Prior to the DRG payment reform, the longitudinal trends for monthly consumable and nursing costs showed a significant increase of 54.62 yuan (*p* < 0.001) and a decrease of 4.53 yuan (*p* < 0.001), respectively. During the implementation month of the DRG payment reform, variations across all cost categories were observed; however, none reached statistical significance. After the reform, nursing costs shifted to a significant upward trend, increasing by 1.48 (*β_1_* + *β_3_*) yuan per month (*p* < 0.001). The introduction of clinical pathway management resulted in immediate and significant shifts in expenditure. During the first month of implementation, immediate significant reductions were observed in total hospitalization costs (651.68 yuan, *p* < 0.001) and nursing costs (19.30 yuan, *p* < 0.05). Furthermore, the subsequent period was characterized by a sustained downward trend in several categories: total hospitalization, consumables, treatment, nursing, and general medical services costs decreased by 85.19, 21.18, 26.36, 4.94, and 13.10 (*β_1_* + *β_3_* + *β_5_*) yuan per month, respectively (*p* < 0.001).

**Table 4:** Interrupted time series analysis of hospitalization costs for patients with impacted teeth.

Cost items	Baseline level (*β*_0_)	Trends prior to the DRG reform (*β*_1_)	Level change during the DRG reform (*β*_2_)	Change in slope after the DRG reform (*β*_3_)	Level change during clinical pathway management implementation (*β*_4_)	Change in trends following the implementation of clinical pathway management (*β*_5_)	Test for autocorrelation		
							*χ* ^2^	lag	*p*
Total hospitalization costs	6009.86 (12.42)^***^	13.16 (0.17)	−447.08 (−0.91)	66.30 (0.84)	−651.68 (−2.95)^**^	−164.65 (−6.53)^***^	0.253	1	0.615
Consumable costs	793.18 (11.99)^***^	54.62 (3.08)^**^	−404.70 (−1.94)	−9.16 (−0.45)	−301.68 (−1.94)	−66.64 (−3.32)^**^	5.625	5	0.018
Treatment costs	3185.63 (10.61)^***^	−26.89 (−0.63)	−48.91 (−0.20)	71.92 (1.61)	−212.23 (−1.48)	−71.39 (−4.35)^***^	3.42	1	0.065
Diagnosis costs	951.90 (7.66)^***^	−11.07 (−0.68)	−19.37 (−0.26)	3.21 (0.19)	−28.23 (−1.31)	0.21 (0.06)	2.935	1	0.087
Medication costs	579.47 (7.99)^***^	3.51 (0.28)	46.78 (0.57)	−9.29 (−0.74)	−59.18 (−1.78)	−5.93 (−1.52)	3.229	1	0.072
Nursing costs	163.64 (22.83)^***^	−4.53 (−4.14)^***^	11.80 (1.31)	6.01 (5.05)^***^	−19.30 (−2.13)^*^	−6.42 (−5.68)^***^	0.65	1	0.420
General medical service costs	336.05 (14.38)^***^	−2.48 (−0.72)	−31.21 (−1.42)	3.17 (0.92)	−29.43 (−1.19)	−13.79 (−4.79)^***^	6.587	2	0.010
Total insurance payments	2749.29 (4.30)^***^	−45.20 (−0.56)	263.83 (0.83)	82.32 (1.00)	−304.94 (−2.20)^*^	−74.87 (−3.75)^***^	3.078	1	0.079
Out-of-pocket expenses	3260.57 (20.37)^***^	58.36 (1.88)	−710.91 (−2.34)^*^	−16.02 (−0.50)	−346.73 (−1.68)	−89.78 (−3.53)^***^	4.285	6	0.039

^*^p < 0.05, ^**^p < 0.01, and ^***^p < 0.001; *z*-scores for the statistical tests are provided in parentheses.

**Figure 8:**
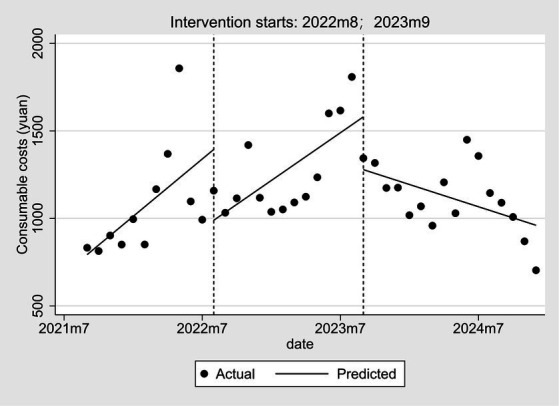
Trends in consumable costs.

**Figure 9:**
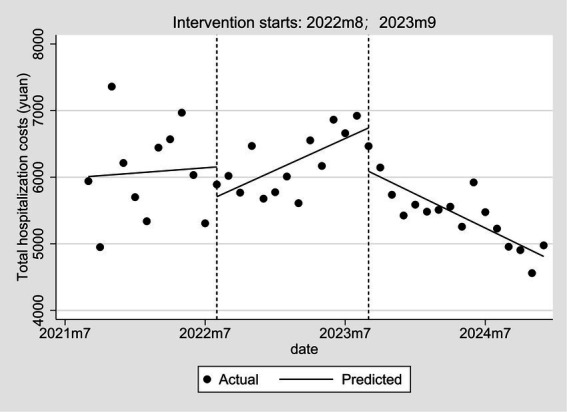
Trends in total hospitalization costs.

The ITS analysis further estimated the monthly trends in payer indicators (Table 4; Figures 8, 9). Regarding the payer structure, changes in total insurance payments before, during, and after the DRG payment reform were not statistically significant. Out-of-pocket expenses decreased significantly by 710.91 yuan (*p* < 0.05) during the month of the DRG reform implementation. However, during the month in which clinical pathway management was implemented, total insurance payments decreased by 304.94 yuan (*p* < 0.05). In the subsequent period, both total insurance payments and out-of-pocket expenses demonstrated significant sustained monthly decreases of 37.75 and 47.44 (*β_1_* + *β_3_* + *β_5_*) yuan, respectively (*p* < 0.001).

### 3.5 Sensitivity analysis

The transition of clinical pathway management under the DRG payment system is a gradual process, and the full manifestation of its policy effects may be subject to a lag. To ensure the robustness of our findings, we performed a lag analysis by setting the two intervention points—DRG policy implementation and clinical pathway management—at a 6-month delay (February 1, 2023 and March 1, 2024, respectively). Based on this reconfiguration, we re-estimated the interrupted time series model for all core healthcare cost indicators. The results showed that the main cost-reduction findings related to the original clinical pathway implementation point were not reproduced under the 6-month delayed intervention specification, which supports the robustness of the effects observed in the main model (see Table 5).

**Table 5:** Sensitivity analysis results of interrupted time series analysis.

Cost items	Baseline level (*β*_0_)	Trends prior to the DRG reform (*β*_1_)	Level change during the DRG reform (*β*_2_)	Change in slope after the DRG reform (*β*_3_)	Level change during clinical pathway management implementation (*β*_4_)	Change in trends following the implementation of clinical pathway management (*β*_5_)	Test for autocorrelation		
							*χ^2^*	lag	*p*
Total hospitalization costs	6097.84 (16.21)^***^	−9.03 (−0.28)	535.12 (1.42)	−49.89 (−1.04)	−23.44 (−0.08)	−42.19 (−0.97)	0.418	1	0.518
Consumable costs	925.22 (9.65)^***^	21.18 (2.50)^*^	28.79 (0.15)	−27.07 (−1.17)	9.09 (0.05)	−30.79 (−0.94)	9.154	1	0.003
Treatment costs	3152.77 (13.92)^***^	−18.28 (−0.92)	451.18 (2.07)^*^	2.85 (0.10)	36.82 (0.27)	−23.29 (−0.85)	2.569	1	0.109
Diagnosis costs	948.40 (9.92)^***^	−11.06 (−1.37)	13.61 (0.28)	−1.30 (−0.16)	45.14 (1.27)	0.20 (0.04)	2.918	1	0.088
Medication costs	574.74 (10.82)^***^	5.53 (1.15)	−26.89 (−0.54)	−19.45 (−2.99)	−34.80 (−0.97)	9.41 (1.81)	1.898	1	0.168
Nursing costs	154.54 (19.62)^***^	−2.22 (−3.30)^**^	32.98 (2.80)^**^	−1.39 (−0.87)	−19.29 (−1.63)	1.22 (0.58)	2.624	1	0.105
General medical service costs	342.16 (18.06)^***^	−4.18 (−2.50)^*^	34.08 (1.19)	−3.69 (−0.81)	−56.96 (−1.54)	1.23 (0.24)	5.083	2	0.024
Total insurance payments	2582.15 (5.28)^***^	−3.49 (−0.08)	406.86 (1.41)	−29.16 (−0.64)	−107.54 (−0.76)	10.14 (0.32)	1.63	1	0.202
Out-of-pocket expenses	3515.69 (13.93)^***^	−5.54 (−0.26)	128.25 (0.49)	−20.73 (−0.62)	84.10 (0.38)	−52.32 (−1.43)	5.992	1	0.014

^*^p < 0.05, ^**^p < 0.01, and ^***^p < 0.001; *z*-scores for the statistical tests are provided in parentheses.

## 4 Discussion

### 4.1 Phased fluctuations and subsequent decline in total hospitalization costs

Total hospital costs for patients with impacted teeth exhibited a phased increase from 5,311.24 yuan to 5,924.73 yuan following the DRG reform. ITS analysis further demonstrated that the trend in total hospitalization costs did not change significantly prior to the implementation of clinical pathway management. While this lack of immediate cost containment contrasts with several studies (13–15), it aligns with findings (16, 17) suggesting that DRG payment reform may be insufficient as an isolated policy instrument. During the initial implementation phase, hospital management and clinical departments underwent a period of adaptation and observation, which likely precluded immediate cost shifts. Furthermore, underdeveloped disease-specific cost accounting may have left hospitals with insufficient data to identify and address cost-control vulnerabilities (18). To maintain financial stability during this transition, hospitals may have also adjusted specific costs upward, leading to higher per-case total hospitalization costs (19).

Conversely, the implementation of clinical pathway management catalyzed a sustained and steady decline in total hospitalization costs. Compared with the post-DRG payment reform period, costs decreased by 840.21 yuan, ultimately falling below pre-DRG reform baseline levels, a finding consistent with Wang et al. (20). This reduction in costs was accompanied by significant improvements in operational efficiency indicators: actual and preoperative length of stay were markedly reduced, while the number of hospital admissions remained stable. These trends suggest that clinical pathway management effectively optimizes medical processes and facilitates cost containment in enhancing medical efficiency. However, its effect on quality improvement needs to be further validated in conjunction with clinical quality indicators.

### 4.2 Sustained decline in cost components following the implementation of clinical pathway management

ITS analysis suggests that clinical pathway management effectively modulates individual cost components. After implementation, consumable, treatment, nursing, and general medical service costs exhibited significant monthly downward trends, aligning with the findings of Liu et al. (15). Notably, while nursing costs rose during the initial DRG adaptation period, the subsequent implementation of clinical pathways appears to have circumvented “cost-shifting” by facilitating coordinated control across consumables, treatment, and nursing cost categories.

As centralized bulk procurement policies for medical consumables have advanced, hospitals have reduced procurement costs through periodic price negotiation mechanisms, such as for absorbable sutures. Physicians have increasingly prioritized cost-effective, high-quality products, gradually refining diagnostic and treatment pathways and thereby reducing the financial burden on patients (21). Under the DRG payment system, hospitals indeed have cost-containment needs, and shortening the length of stay represents an effective pathway to cost reduction. In this study, after the implementation of clinical pathway management, the actual length of stay decreased from 4 days to 1 day, and the preoperative length of stay decreased from 2 days to 0 days. This substantial reduction in hospital stay implies that waiting time and postoperative observation time were curtailed, with associated fixed costs—such as bed fees and basic nursing fees—being correspondingly saved. It should be noted that treatment costs still constitute a significant proportion of total costs and remain higher than levels observed prior to the DRG payment reform, which may be associated with policy support for the value of technical labor (22). Impacted tooth extraction with flap reflection is a technically demanding procedure that primarily relies on specialized medical expertise. The state has repeatedly emphasized in recent years that medical service price adjustments should prioritize items that reflect technical labor value, such as treatment and surgical procedures. Therefore, the relatively high and sustained level of treatment fees, remaining above pre-reform levels, may to some extent reflect reasonable compensation for the technical expertise of healthcare professionals.

### 4.3 Mitigation of patient financial burden

From a health insurance perspective, the implementation of clinical pathway management may have positive implications for both the insurance fund and patients, as both total insurance payments and out-of-pocket expenses exhibited downward trends following its introduction. Our results showed that during the DRG reform implementation month, only out-of-pocket expenses decreased significantly, whereas after the implementation of clinical pathway management, both total insurance payments and out-of-pocket expenses transitioned to a downward trajectory. Compared with the pre-DRG reform baseline, total insurance payments increased by 12.31% following the implementation of clinical pathway management, while out-of-pocket expenses decreased by 15.52%. These findings align with prior research (15, 21) suggesting that standardized clinical protocols effectively regulate provider behavior to reduce patient costs (23). Moving forward, the leverage of health insurance funds should be continuously utilized to optimize cost structures through the combined implementation of payment reform and clinical pathway management. Such integration is essential for curbing medical cost escalation, ensuring the efficient allocation of healthcare resources, and enhancing the overall value and accessibility of healthcare services for residents (24). However, it should be noted that cost optimization does not necessarily equate to quality improvement. Future research should incorporate clinical quality indicators and adopt a multicenter design to comprehensively evaluate the combined effects of these policies.

## 5 Innovation and limitations

This study examined impacted teeth, a common but underexplored oral surgery condition, and assessed the cost-control effects of integrating DRG payment with clinical pathway management. The results indicate that clinical pathway management, as a refined management tool, serves as a complement to DRG payment in terms of cost containment and efficiency improvement. However, several limitations warrant consideration. First, the data were derived from a single tertiary institution, which may limit the generalizability of the results; subsequent research should utilize multicenter datasets to account for regional disparities in healthcare standards. Second, this analysis does not incorporate healthcare quality indicators; future studies should include metrics such as unplanned readmission and complication rates to provide a more comprehensive assessment of policy impact. Finally, this study adopted an interrupted time series quasi-experimental design without a concurrent control group, and it is difficult to completely exclude the confounding effects of concurrent policies, such as centralized bulk procurement of medical consumables, on the observed cost reductions. Consequently, these findings should be interpreted as demonstrating a significant association rather than a definitive causal relationship.

## Data Availability

The original contributions presented in the study are included in the article/Supplementary material, further inquiries can be directed to the corresponding author.
